# Supplementary material for: Are carbon emissions trading and green financial instruments synergistic? -Comprehensive quantitative research based on content analysis
Source: PLoS One. 2024 Mar 7;19(3):e0298601. doi: 10.1371/journal.pone.0298601 (PMC10919601; doi:10.1371/journal.pone.0298601)
Supplement: S1 Data — (ZIP) [file pone.0298601.s001.zip › Data/地级市Z_F工作报告.zip!/║■▒▒/╗╞╕╘╩╨/└┤╫╘╣1⁄2╓┌║┼í╛┬φ┐╦╩2╛▌═°í┐_2004.docx]

各位代表：

　　现在，我代表市政府向大会作政府工作报告，请予审议。并请市政协各位委员和列席人员提出意见。

　　一、2003年工作回顾

　　过去的2003年是不寻常的一年。在市委正确领导下，市政府团结带领全市人民，认真落实市二届人大二次会议确定的各项目标和举措，经受住了“非典”疫情的严峻考验，在全面建设小康社会道路上迈出了比较坚实的一步。

　　——紧紧抓住第一要务，促进了市域经济全面发展。坚持“全党抓经济，重点抓工业，突出抓招商，着力抓环境”，实行总量扩张与结构调整一起抓，基本做到了速度、质效和后劲相统一。全市完成GDP388亿元，比上年增长7.5%。完成财政收入24亿元，比上年增长6%。农业“五带十区”工程全面启动，特色农业和优质农产品比重明显上升，农产品商品率和加工转化增值率明显提高，农业增加值增长3.4%，是近5年来的最高增幅。工业产销和质效同步上升，工业增加值增长9.9%，利润增长11.9%，工业综合经济效益指数达到143.9%，为建市以来最好水平。第三产业增加值增长7.8%;社会消费品零售总额达到136.5亿元，增长9.4%;金融机构年末存贷款分别达到256.8亿元和202.7亿元，比年初增长20.4%和8%;旅游、教育、房地产、现代通信、社区服务等新兴产业保持了较快的发展势头。

　　——扎实推进各项改革，增强了经济发展的体制活力。以民营化为取向，以破产重组为重点，企业改革实现新突破，国改民的比例达到80%以上，非公有制经济比重提高到70%左右。农村税费改革深入推进，农民负担继续减轻，乡村两级组织和农村义务教育运转基本正常，化解村级债务工作初步展开。行政审批制度改革不断深入，取消了一批审批和收费项目，行政服务中心、财会核算中心、政府采购中心、建设工程招投标中心和土地储备交易中心全面启动并规范运作。积极落实省委、省政府关于加快县域经济发展的战略部署和政策措施，县市经济“一主三化”进程明显加快。推进财政支出管理改革，对公检法司等部门实行了比较规范的部门预算。乡镇配套改革、国有农场改革、机关事业单位机构和人事制度改革都取得新的进展。

　　——全面启动“三个百亿投入工程”，培植了一批新的经济增长点。按照市委关于在“十五”后三年完成能源、交通和招商引资各投入100亿元的要求，坚持把投资作为加快发展的第一支撑，把改善环境作为投资创业的第一要素，招商引资和重大项目建设赢得前所未有的好势头。全市完成固定资产投资107亿元，比上年增长12%，其中50万元以上项目投资增长39.3%。全市实现外商投资4942万美元，比上年增长52.6%;引进国内资金到位40.3亿元，其中投资亿元以上项目17个，千万元以上项目199个，一批知名企业和品牌相继落户黄冈。积极争取国家投资项目，黄冈大别山电厂、白莲河抽水蓄能电站已成立公司并开始运行，江北一级公路、干线路网、县乡公路、大中型水库整险、遗爱湖综合治理等重点建设工程和黄冈天然气、大别山生态旅游等重大项目前期工作进展顺利。

　　——积极加快城镇建设和发展，提升了黄冈的城市形象。坚持抓市区带全市、抓本级带下级，市区主要经济指标完成情况好于上年、好于全市平均水平，市直招商引资、国企改制、工业园区建设在全市起到了示范作用。市区十件大事大多完成或超额完成了年度工作任务。成功承办了省运会篮球比赛和全省农民秧歌比赛，举办了第三届东坡赤壁文化旅游节暨首届全市运动会、大别山农民科技节、香港招商洽谈会等节会活动，扩大了黄冈的影响和知名度。加快实施城镇化战略，城乡规划、建设、管理水平进一步提高。市区投入城建资金5400万元，新建和配套了一批促进投资创业和改善人民生活的市政设施;实施“一把手”工程，建立健全长效机制，深入推进“门前四包”，加强社会治安综合整治，取缔麻木和摩托车非法载客营运，市区形象焕然一新。各县市累计完成城建投资7.2亿元，实施项目1210个，县市城区、中心镇、口子镇和中心村建设步伐加快，城乡面貌有了新变化。

　　——着力解决事关群众切身利益的问题，确保了人民生活有所改善。高度重视“非典”防治，加强组织领导，实行严防死守和科学防治，有效控制了“非典”疫情扩散和蔓延。层层建立就业再就业责任制，千方百计改善就业环境和拓宽就业渠道，全市发放再就业优惠证2.7万个，净增就业岗位1.8万个，安置下岗失业人员2.8万人，城镇登记失业率控制在4.4%以内。加强社会保障体系建设，严格按规定将社保资金纳入财政预算，确保了国企下岗职工基本生活费和离退休人员基本养老金按时发放;依法扩大社会保险覆盖面，全市养老、医疗、失业保险分别增加1.4万人、3.6万人和6700人;健全城市最低生活保障制度，全市纳入低保的城镇居民达到17.6万人，基本实现应保尽保。加快“福星工程”建设，集中供养“五保”对象1.4万人。大力实施扶贫济困工程，全市未解决温饱人口减少到45万人以下，温饱巩固率达到91%。人民生活水平继续得到提高，全市城镇居民人均可支配收入达到5100元，农民人均纯收入达到2204元，城乡居民人均储蓄存款达到2920元，分别比上年增长4%、3.4%和38%。

　　——切实加强政治文明和精神文明建设，促进了社会事业全面进步。坚持依法行政、从严治政，完善了政府目标管理责任制和行政执法责任制。自觉接受人大依法监督和政协民主监督，虚心征求和听取人大代表、政协委员对政府工作的意见，人大议案、代表建议和政协建议案、委员提案的办结率、满意率进一步提高。大力“打造平安黄冈，创建安全城市”，及时妥善处理群众来信来访和群体性事件，深入开展“严打”斗争和同“法轮功”等邪教组织的斗争，高度重视和做好安全生产工作，维护了社会稳定。积极开展国防教育和“双拥”活动，密切了军政、军民关系。加大领导干部廉洁自律、经济责任审计、大案要案查处和源头治腐工作力度，廉政建设进一步加强。深入开展“三个代表”重要思想学习教育活动、群众性精神文明创建活动和市直机关“三评一促创五好”活动，干部群众思想政治素质和城乡文明程度有了新提高。大力实施科教兴市战略，科技成果转化率、科技进步对经济增长的贡献率继续上升。扎实推进教育改革和素质教育，调整优化教育布局，各类各层次的教育进一步发展。积极支持文化事业和文化产业发展，社会科学、文学艺术、新闻出版、广播影视、史志修编等事业繁荣活跃。加强公共卫生建设，健全城乡预防、保健、医疗网络，改善了居民就医和卫生条件。全民健身活动蓬勃开展，竞技体育水平继续提高。加强人口与计划生育工作，大力整治出生婴儿性别比失衡问题，保持了低生育水平。深化土地使用制度改革，国土资源管理水平和使用效益同步提高。加强环境保护和综合整治，遏制了生态环境恶化趋势。民族宗教、外事侨务、对台、人防、统计、档案、保密、老龄、残疾人等各项工作都取得新的成绩。

　　各位代表，过去一年成绩来之不易。这是在党中央、国务院、省委、省政府和市委正确领导下，全市人民团结拼搏、发奋努力的结果;是历任黄冈领导打下良好基础和市人大、市政协有效监督与关心支持的结果;是驻黄部队、武警官兵、各民主党派、工商联、社会各界积极参与的结果;是上级有关部门、外地黄冈籍人士、国内外各界朋友尤其是来黄冈投资的外地客商鼎力相助的结果。在此，我代表市政府，向全市人民，向所有关心支持黄冈建设的各级领导和海内外朋友，表示由衷的感谢和崇高的敬意!

　　各位代表，我们也清醒地看到，前进中还面临许多亟待解决的问题。主要是：经济增长速度偏低，部分主要指标低于全国、全省平均水平，没有圆满完成年初预期目标;市场主体尤其是本地创业主体活力不足，民营经济和外向型经济发展不快，具有竞争力的品牌、企业和企业家严重短缺;各级财政收支矛盾突出，地方一般预算收入增长乏力，城乡居民收入增幅较低;就业再就业压力增大，社会保障体系不够健全，部分下岗失业人员、城镇低收入居民和贫困地区群众生活还比较困难;社会治安和安全生产隐患不少，影响社会稳定的因素增多;政府职能转变的步子不够大，有的政府工作人员还存在官僚主义、形式主义、作风粗暴和腐败行为。对这些问题，我们一定高度重视，在今后的工作中切实加以解决。

　　二、2004年的奋斗目标和工作重点

　　今年政府工作的指导思想是：以邓小平理论和“三个代表”重要思想为指导，全面贯彻党的十六大、十六届三中全会和中央、全省经济工作会议精神，牢固树立全面、协调、可持续的科学发展观，坚持“三改一培育”(改善环境、改革体制、改进作风和培育市场主体)和“一主三化”(以民营经济为主体，推进工业化、农业产业化和城镇化)的工作方针，坚持“全党抓经济，重点抓工业，突出抓招商，着力抓环境”的工作思路，坚持“从一抓起，实干兴市”的工作方略，大力实施项目立市、工业兴市、人才强市、改革活市、依法治市战略，全面推进三个百亿投入工程、农业五带十区工程、扶贫济困工程、文明创建工程、干部人才素质工程，进一步提高民营经济、工业经济、城市经济、外向型经济和地方一般预算收入占GDP的比重，力保全市经济和社会发展主要指标增幅不低于全省平均水平，更好地实现就业增加、企业增效、财税增长、居民增收，促进物质文明、政治文明和精神文明协调发展。主要预期目标是：GDP增长9%;地方一般预算收入增长10.5%;全社会固定资产投资增长15%，社会消费品零售总额增长10%;外贸出口增长12%，外商投资增长20%;城镇居民人均可支配收入和农民人均纯收入分别增长6%和5%;全年净增就业岗位2.2万个，城镇登记失业率控制在4.4%以内;人口自然增长率控制在6‰以内。

　　确定并实现上述目标，一是有比较有利的国际国内环境。世界经济逐步回升和结构调整加快，国际投资、贸易进一步看好中国，为我们扩大开放提供了有利条件。国家继续实施积极的财政政策和稳健的货币政策，增加对农业和农村的投入，加大扶贫济困力度;沿海产业加速向中西部梯度转移，武汉城市经济圈正在崛起，为我们加快发展带来了新的机遇。二是有比较良好的现实发展基础。我市通过狠抓招商引资和重大项目，已开工和今年可望开工的过亿元项目有30多个，一批新的经济增长点正在形成;通过大力推进结构调整和新型工业化，一些特色支柱产业和骨干企业正在壮大;通过加大“一主三化”和国企改革力度，县域经济尤其是民营经济日趋活跃;通过县市领导班子成功换届，干部群众加快发展的自觉性和积极性进一步增强。三是有十分紧迫的自身发展要求。黄冈是个欠发达地区，最主要的问题仍然是发展不够，在当前全国特别是周边地区竞相加快发展的大趋势下，我们要解决社会经济生活中的诸多矛盾和问题，逐步缩小同全国、全省尤其是发达地区的差距，实现全面建设小康社会的宏伟目标，必须强化忧患意识和竞争意识，千方百计保持一个较快的增长速度。

　　今年政府工作重点抓八个方面：

　　(一)进一步突出招商引资，加大项目投入力度

　　继续把招商引资作为谋发展的第一举措。坚持以提高质效为中心，以工业项目为重点，实行内资外资一起引，引进项目、资金、品牌、技术、人才、管理并重，进一步完善领导带头、部门联动、政策引导、重奖激励、全民参与的招商格局，强化市场化、企业化、专业化招商和以安商促招商，在切实抓好汇源、天颐、太子奶、中水集团、神龙纺织、华新水泥、台湾顺天堂、美国杜邦、比利时高格等现有招商引资项目跟踪服务的基础上，再集中力量新引进一批投资大、档次高、带动力强的知名企业和品牌，力争今年全市招商引资突破50亿元。

　　千方百计加强重点项目建设。结合招商引资突破性地加大工业项目投入，确保重点项目中工业项目占三分之一以上，全社会固定资产投资中工业投资占三分之一以上。继续抓好基础产业、基础设施和城乡公共事业项目建设，能源方面，重点抓好黄冈电厂、大别山电厂、白莲河抽水蓄能电站和黄冈天然气工程等项目;交通方面，重点建设阿(内蒙阿荣旗)深(圳)、武(汉)英(山)两条高速公路黄冈段，江北公路及其连接线、红(安)武(汉)、阳(逻)黄(州)三条一级公路，以及国省道二级公路190公里和通乡通村油路750公里;农林水利方面，重点建设名优农产品商品基地、退耕还林、中德林业合作项目、大别山火险综合治理、农村人畜饮水解困、16座大中型水库整险加固和白莲、明山、金檀三个灌区节水改造等工程;公共卫生方面，重点建设市县两级疾病防控中心、紧急救援中心和市传染病医院、县市传染病区、乡镇发热门诊项目;生态环境方面，进一步抓好大别山生态旅游和遗爱湖综合治理等重点项目。继续加强同中央和省有关部门的衔接，争取更多的项目进入国家投资计划。加强重大项目建设的组织协调，严格落实项目建设“四制”，确保工程建设质效。

　　下大力激活多元投资主体。在积极争取国家投资、全力扩大招商引资的同时，进一步加快要素市场建设，扩大直接融资比重;加强同金融部门的沟通与衔接，积极推荐优质项目，配合处理不良债务，更多更好地利用银行信贷投资;按照“非禁即入”的要求，大胆放开投资领域，吸引和集合民间资本搞建设，力争民间投资有较大幅度的增长。

　　锲而不舍地改善创业和发展环境。围绕打造“低成本、快回报、高诚信、优服务、零障碍”环境优势，进一步加强行政服务中心、经济发展环境投诉中心、综合招投标中心和电子政务建设，完善办事规则和程序，加大破坏经济环境案件的查处力度，努力为各类市场主体提供周到、便捷、优质、高效的服务。建立健全政府、企业和个人信用体系，大力开展A级信用城市、最佳金融信用县市、信用企业、信用农户和信用金融机构的创建活动，打造信用黄冈。继续整顿和规范市场经济秩序，严厉打击制假售假、商业欺诈等违法犯罪行为;加强市场监管，深入开展医药、食品等各类专项整治;坚决打破地区封锁和行业垄断，加快构建统一、公平、规范、有序的市场体系。

　　(二)毫不动摇地重点抓工业，加快走新型工业化道路

　　认真制定和实施新型工业化规划。结合启动“十一五”计划编制和抓好全国第一次经济普查，加快出台和完善全市新型工业化规划。处理好工业化与信息化、国际化、市场化、城市化的关系，注重通过加快信息化、应用高新技术来带动工业化;通过融入国际化、提高开放度来推进工业化;通过运用市场机制引资金、换技术、搞招商来加速工业化;通过推进城市化和区域经济一体化实现产业聚集，积极呼应和融入武汉城市圈建设，在更大范围实现专业化分工协作，培植和形成大产业。在充分发挥市场基础作用和企业主导作用的同时，发挥好政府引导作用，重点抓好产业政策制定、园区规划建设、对外招商引资和启动民间投资等工作。

　　着力培植支柱产业、骨干企业和精品名牌。按照“改造提升传统产业、做大做强支柱产业、培育发展高新产业”的思路，加大工业技改投资和招商引资力度，着力培植轻工食品、医药化工、纺织服装、建筑材料、机械电子五大支柱产业，培植一批利税过千万元、过亿元的工业小巨人，发展一批“专、精、特、新”的中小企业，形成一批具有地方特色和核心竞争力的工业骨干企业、农业产业化龙头企业和精品名牌。

　　继续加快各类开发区和工业园区建设。按照“科学规划、合理布局、基础先行、滚动开发”的原则，以发展工业和招商引资为重心，抓好各类开发区的定位和整合，推动开发机制和管理体制创新，建设一批服务优良、管理规范、功能完善、产业配套、优势明显的经济开发示范区。进一步加大工业园区建设力度，支持老城区企业往园区搬迁改造，吸引外地客商到园区投资办厂，鼓励本地民间资本向园区集中创业，促进资金向产业、企业向园区聚集，以园区为依托打造产业集群。

　　努力提高黄冈产品和服务的市场占有率。坚持统筹开拓城乡市场、工业品与农产品市场、市内与市外市场、国内与国外市场，积极参加和举办各类行之有效的节会活动，通过多种渠道拓展黄冈产品市场空间，争取更多的黄冈产品进入国际采购网络和国内大超市。深化物流企业改革，大力发展连锁经营、物流配送、电子商务等现代营销方式和流通组织形式。大力开拓服务业市场，重点抓好旅游、教育、房地产、现代通信、文娱健身、社区服务等新兴消费热点的培植。启动中国优秀旅游城市创建工作，加快打造精品景区，强化旅游宣传促销、旅游产品开发和旅游行业创优，扩大旅游经济规模。充分发挥教育优势，推动教育消费及相关产业的发展。加大房地产开发和监管力度，放开搞活土地和住房二级市场，扩大城乡居民住房消费。

　　(三)以农民增收为核心，促进农业和农村经济发展

　　高度重视“三农”工作。认真贯彻中央1号文件精神，坚决落实“多予、少取、放活”的方针，坚持以农民增收为核心，以深化改革为动力，以调整优化结构、推进产业化经营为主线，以统筹城乡发展、打破城乡二元结构为目标，力争全市农业、农村经济和社会面貌有较大改善。

　　大力实施“五带十区”工程。坚持用工业理念谋划农业，走以龙头企业带大产业、以产业组织农民的路子，进一步盘活盘强现有龙头企业，下大力引进国内外知名加工型、流通型龙头企业，不断提高我市农产品转化增值能力。围绕龙头企业，发挥资源和产业优势，集中连片建设高效优质农产品基地，推进区域化、规模化、特色化种养。完善龙头企业与基地和农户之间的利益机制，促进企业和农民双利双赢。

　　稳定粮棉油生产能力。增强粮食安全意识，发挥粮棉油生产的综合优势。在遵循市场规律、尊重农民意愿的前提下，发挥政策引导和激励作用，调动和保护农民从事粮棉油生产的积极性，重点引导农民在消灭抛荒田地和提高单产、提高复种指数、提高产品质量上下功夫。认真落实国家对粮食生产的扶持政策，确保粮食主产区种粮补贴直接补给种粮农民，抓好连片万亩以上的标准化高产农田示范区建设。

　　增加对农业和农村经济投入。加大农村水、电、路等基础设施和文、教、卫等公共设施建设力度，不断改善农业和农村生产生活条件。加大农科教统筹和投入力度，抓好对农民的科技培训。加强农产品检验检测体系和疫病防治体系建设。落实对农业和农民的信贷支持措施，增加农户小额信用贷款和农户联保贷款。

　　积极发展非农产业。在抓好乡镇企业改革发展、加快壮大农村个体私营经济的同时，切实做好农村劳动力输出的组织、引导、管理和培训等工作，加快建立以农民工就业信息服务、就业培训服务、就业中介服务和维权服务为重点的农民工跨地区就业服务体系，促进“打工经济”持续健康发展。

　　加大扶贫开发力度。认真总结推广开发式扶贫、搬迁扶贫、对口帮扶等工作经验，完善扶贫开发机制，多渠道增加扶贫开发投入，改革扶贫项目管理体制，落实更有效的优惠政策和扶持措施，促进扶贫开发工作再上新水平。

　　(四)着眼于培育市场主体，深度推进改革开放

　　加快市域经济民营化步伐。进一步加大企业产权制度改革力度，争取今年内市及市以下所属国有和集体工商企业全面实现“三个退出”，成为民有民营的市场主体，使民营企业成为黄冈经济发展的主力军。推进国有资产管理体制改革，整合政府性资源，实行集中管理、统一经营、市场运作。取消一切对民营经济的歧视和限制，在市场准入、土地征用、融资贷款、税收征管等各个方面，给予本地民间资本和外来投资企业以平等国民待遇，提供同等政策优惠和支持服务，促进民营经济做大做强。

　　扎实抓好农村各项改革。稳定和完善家庭承包经营制度，依法保障农民对土地承包的各项权利。深化农村税费改革，重点抓好适当降低农业税税率和取消农业特产税等有关工作，落实财政转移支付补助政策，积极化解村级债务，加强涉农收费监管，确保农民人均负担再减少10元以上。全面推进乡镇综合配套改革，实现乡镇机构编制管理法定化、刚性化。积极推进国有农场改革，实行国有农场管理体制及内部运行机制改革与职工养老统筹、农村税费改革“三改”联动，逐步建立起有利于国有农场更快更好发展的管理体制和运行机制。

　　积极稳妥推进事业单位改革。全面引入竞争机制，变“养人”为“养事”。严格清理和规范行政执法类事业单位，重新定编定岗，精干机构设置;调整重组社会公益性事业单位，改革事业费投入方式和管理方式;加快生产经营类、中介服务类和技术服务类事业单位产权制度改革，使之成为自负盈亏的市场主体。争取今年内基本完成事业单位改革任务。

　　深化财政管理体制改革。积极转变生财用财聚财观念，狠抓财源建设，加强收入征管，合理安排支出。按照统一部门预算编制范围、统一人员编制口径、统一公用经费口径、统一使用预算内外资金、统一国库集中支付、统一公共财政收支科目的原则，实行“一个部门一本预算”。大力推进国库集中收付制度、政府采购制度、收支两条线和预算外资金管理制度等改革。

　　扩大对外出口和经济技术交流。坚持市场多元化和以质取胜战略，调整优化出口商品结构，加大纺织服装和医药化工等重点产业和产品的出口力度，加快培育高技术含量、高附加值出口商品群。认真落实国家鼓励出口的各项政策。深化外贸体制改革，积极引导外贸出口从收购制向代理制转变，培植多元出口经营主体。鼓励支持有条件的企业到外地投资办厂、承接工程、开展加工贸易和资源开发，带动产品、技术和劳务输出。充分发挥外事、侨务、对台部门的作用，扩大对外交流与合作。

　　(五)着力高昂市区龙头，加速城乡一体化进程

　　继续集中精力发展市区。坚持把市直工作重点转到抓本级带下级上来，确保市区主要经济和社会发展指标增幅高于全市平均水平。以壮大市直工业为主导，一手抓改革转制和技术改造盘活老产业、老企业，一手抓招商引资和集合民资发展新产业、新企业，突破性地提升市区经济规模和档次。继续加快省级高新技术开发区尤其是东湖工业园和西湖工业园的建设，倾力打造几个有特色的工业板块和产业集群。以贸易广场和步行街建设为重点，进一步繁荣市区商贸服务业。大力发展市区旅游、教育、房地产、社区服务和城郊现代农业。加大市区建设投入力度，力争今年市政建设投资突破2亿元。在继续完成过去几年市政府承诺的大事实事部分未竟事项的基础上，今年再努力新办十件实事：一是争取新上3个过亿元的投资项目;二是抓好工业园区“三通”配套建设;三是新建一条投资过亿元的步行街;四是完成南湖路(即江北公路接线)、中环路等6条道路的新建和东坡大道、黄州大道等7条街巷的配套;五是办好黄冈中学百年校庆并启动该校易地新建;六是完成东湖泵站建设;七是抓好滨江公园续建工程;八是新增就业岗位3000个以上;九是完成文化中心及“职工之家”配套建设;十是完成市区48座公厕的水冲式改造。

　　大力提升县域经济实力和竞争力。全面落实扶持县域经济发展的各项政策，尽可能赋予县市更多的发展自主权。加速“一主三化”进程，加快构筑县域经济以民营为主体的发展格局，加快形成一批具有竞争优势的支柱产业和骨干企业。继续推进县市区“八个一”工程建设，即：一个外商投资过100万美元的项目，一个外来投资过亿元的项目，一个民营资本过1000万元的企业，一个高标准的工业园区，一个城市公益事业亮点工程，一个管理规范的文明社区，一个连片万亩以上的优质高效农产品基地，一个有带动力的农业产业化龙头企业。进一步建立完善考核奖惩机制，在各县市区之间形成你追我赶、竞相发展的氛围。

　　加强城镇规划、建设、管理和经营。按照“统一规划、产业先行、多元投入、配套建设、高效管理、特色建城”的思路，完善“一心(黄冈市区)两翼(麻城、武穴)三带(长江沿岸、京九沿线、大别山腹地)”的城镇体系，高标准抓好城镇规划编修，进一步强化城镇规划管理;以市县城区和中心镇、口子镇为重点，加快城镇基础设施配套建设，增强城镇整体功能;深入推进“门前四包”，加大洁绿亮美力度，提高城镇管理水平;大力推进城市经营，加快城镇投融资体制改革、土地使用制度改革、户籍管理制度改革和公用事业单位民营化改革，打破城乡樊篱，促进城乡人财物自由流动，走以城带乡、城乡一体、共同繁荣的新路子。

　　(六)高度关注民生问题，尽可能给群众更多实惠

　　坚持把就业作为民生之本。实施积极的就业政策，改善就业环境，拓宽就业再就业领域和渠道。大力发展劳动密集型产业和个体私营经济，鼓励下岗失业人员灵活就业和自主创业。认真落实扶持就业再就业的各项政策措施，重点落实好财政扶持、信贷支持、税费减免等政策。健全再就业援助制度，政府出资创造的公益性岗位，优先安置就业困难人员。完善就业服务体系，强化下岗失业人员再就业培训。今年全市再发放再就业优惠证2.8万本，安置下岗失业人员再就业2万人。建立和完善劳动关系协调机制，加强劳动用工监察、工资支付监管和劳动争议处理工作。统筹做好复员转业军人安置、大中专毕业生、城镇新成长劳动力和农村富余劳动力就业工作。

　　加强社会保障体系建设。继续抓好“两个确保”和城市“低保”工作，严格将社保资金纳入财政预算，增加社会保障支出，规范社保资金管理，确保国有企业下岗职工基本生活费和离退休人员基本养老金按时足额发放，确保失业人员按规定足额享受失业保险金待遇，确保符合条件的城镇贫困人口全部纳入最低生活保障范围。进一步扩大养老、失业、医疗、工伤、生育保险的覆盖面，新增扩面4.65万人;着力提高社会保险费的征缴率，确保缴费人数达到参保人数90%以上。

　　努力提高人民健康水平。积极推进卫生改革和发展，抓好突发公共卫生事件应急处理体系、疾病预防控制体系、医疗救治体系、环境卫生体系和卫生执法监督体系建设。进一步健全非典、艾滋病、血吸虫病等重大传染病防治工作的长效机制。积极开展全民健身活动，进一步提高竞技体育水平，加快公共体育设施的建设与配套，发展体育产业。认真落实妇女儿童发展纲要，加强老年人权益维护，关心支持残疾人事业。

　　加快“创建平安黄冈，打造安全城市”。认真落实社会治安综合治理各项措施，强化治安基础防范工作。深入开展“严打”斗争，依法严厉打击各种刑事犯罪、经济犯罪和黑恶势力，提高大案要案侦破率。坚决查禁各种社会丑恶现象，净化社会环境。加强公安、司法和国家安全部门建设，加强反恐斗争和打击“法轮功”等邪教组织的斗争，防范和打击境内外敌对势力的渗透和破坏活动。认真落实民族宗教政策，积极维护民族团结，依法管理宗教活动。做好人民群众来信来访和人民调解工作，完善化解人民内部矛盾的工作机制，积极预防和妥善处理突发性事件和群体性事件。高度重视和抓好安全生产，防止重特大事故发生。

　　(七)实施科教兴市、人才强市，协调发展各项社会事业

　　促进科技与经济紧密结合。继续加强“科技致富工程”建设，充分发挥科技特派员的作用，抓好优质农产品及其加工技术的推广应用，加快农村科技信息网建设，提高农民科技素质和致富能力。加强以企业为主体的技术创新体系建设，引导企业增加科技投入，增强自主创新能力。加强科技市场管理，支持民营科技企业发展。加快高新技术开发区和高新技术产业带建设，促进科技成果向现实生产力转化。

　　坚持把教育放在优先发展的战略地位。巩固完善以县为主的农村义务教育管理体制，依法加大对教育投入，大力发展高中教育、职业技术教育和成人教育，加快发展高等教育，积极发展民办教育。加强教师队伍建设，按政策规定妥善处理好民办教师问题，切实提高教师的思想和业务素质。继续积极支持黄冈师院、黄冈职院和黄冈中学建设与发展。

　　大力实施人才强市战略。坚持人才资源是第一资源，实行培养开发人才和引进人才并重，重点吸引高层次人才和紧缺人才。落实知识、技术、管理等要素参与分配政策，抓好企业经营者年薪制实施，重奖有突出贡献的各类人才。抓紧制定人才资源规划，重点做好制定政策、整合力量、营造环境等工作，努力做到用事业造就人才、用环境凝聚人才、用机制激励人才、用法制保障人才。

　　认真贯彻“三大基本国策”。严格执行计划生育政策，进一步完善和落实“一把手”负总责和目标管理责任制，加强和改进乡村技术服务体系建设，进一步重视解决出生婴儿性别比例失衡问题，确保人口安全。依法保护和合理利用土地、水、矿产等宝贵资源，健全资源有偿使用制度，强化资源执法监督，提高资源综合利用率。加强环境综合治理，认真落实环保“第一审批权”，严格执行环境影响评价和“三同时”制度，抓好一批重点生态环境工程建设，积极推行清洁生产，发展循环经济，促进人与自然和谐发展。

　　加强思想道德和文化建设。坚持用邓小平理论和“三个代表”重要思想武装广大干部群众，积极开展群众性精神文明创建活动，努力在全社会形成共同的理想信念和精神支柱。认真贯彻《公民道德建设实施纲要》，深入开展社会公德、职业道德和家庭美德教育，在全社会形成良好的基本道德规范。加强科普宣传教育，破除封建迷信，倡导科学精神和文明健康的生活方式。围绕建设文化强市，深化文化体制改革，加强城乡文化设施建设，完善文化公益事业发展政策，进一步繁荣文化事业和文化产业。加快新闻出版、广播影视业的改革发展步伐。抓好文物保护、史志修编和档案、保密等工作。

　　积极支持军队和国防建设。深入开展国防教育，健全国防动员体系。关心支持驻军和武警部队建设，加强民兵预备役和人民防空工作。深入开展“双拥”和军民共建活动。做好军队转业干部、复员退伍军人接收安置和优抚工作。

　　(八)坚持依法行政、从严治政，切实加强政府自身建设

　　努力建设法治政府。严格按法定权限和程序行使权力、履行职责，力求不失职、不超权，把政府各项行政行为纳入法制化轨道。认真实施《行政许可法》，加大行政审批制度改革力度，继续减少并规范行政审批行为。加快改革和理顺行政执法体制，全面落实行政执法责任制，启动相对集中行政处罚权。深入推进政务公开，进一步增强政府工作透明度。自觉接受市人大及其常委会的法律监督，高度重视人民政协的民主监督和参政议政，充分发挥工会、共青团、妇联等人民团体的桥梁和纽带作用，推进政府决策科学化、民主化。

　　努力建设服务政府。按政企、政事分开和精简、统一、效能的原则，进一步转变政府职能，调整机构设置，理顺部门分工，改革政府调控和监管方式，切实把政府职能转到经济调节、市场监管、社会管理和公共服务上来。完善公务员制度，建设高素质的公务员队伍。进一步强化公仆意识和服务意识，满腔热忱地为基层和群众服务、为市场主体和纳税人服务。坚持解放思想、实事求是、与时俱进的思想路线，坚持讲真话、出实招、办实事、求实效，坚决反对官僚主义、形式主义，大力精简会议、文件和应酬，扑下身子多做打基础、管长远的工作，力求干一件成一件，使各项工作经得起群众的评判和历史的检验。大兴调查研究之风，经常深入基层、深入实际、深入群众，及时解决好群众关心的热点难点问题，真正做到权为民所用、情为民所系、利为民所谋。

　　努力建设廉洁政府。大力弘扬“两个务必”的优良传统，坚持艰苦创业、勤俭办一切事业，坚决制止不切实际的“形象工程”、“政绩工程”，坚决反对各种滥用民力、奢侈浪费的行为。认真贯彻中纪委三次全会提出的关于领导干部廉洁自律的“四大纪律、八项要求”，全面落实党风廉政建设责任制，加强行政监察和审计监督，加强对政府机关工作人员的严格教育、严格管理，加强部门和行风建设，加大源头治腐和大案要案查处力度，以卓有成效的反腐倡廉成果取信于民。

　　各位代表!今年改革和发展的任务十分艰巨。我们要紧密地团结在以胡锦涛同志为总书记的党中央周围，高举邓小平理论和“三个代表”重要思想伟大旗帜，深入贯彻党的十六大和十六届三中全会精神，在市委统一领导下，紧紧依靠全市人民，以更饱满的热情、更昂扬的斗志、更务实的作风，抓抢机遇，开拓创新，奋力“在全省市州中争进位，在全国老区中创一流”，为全面建设小康社会、加快富民强市步伐而努力奋斗!
